# Negative consequences associated with dependence in daily cannabis users

**DOI:** 10.1186/1747-597X-2-3

**Published:** 2007-01-10

**Authors:** Alison Looby, Mitch Earleywine

**Affiliations:** 1 University at Albany, State University of New York, Department of Psychology, SS369, 1400 Washington Avenue, Albany, NY, 12222, USA

## Abstract

**Background:**

Cannabis is the most widely consumed illicit substance in America, with increasing rates of use. Some theorists tend to link frequency of use with cannabis dependence. Nevertheless, fewer than half of daily cannabis users meet DSM-IV-TR criteria for cannabis dependence. This study seeks to determine whether the negative aspects associated with cannabis use can be explained by a proxy measure of dependence instead of by frequency of use.

**Results:**

Over 2500 adult daily cannabis users completed an Internet survey consisting of measures of cannabis and other drug use, in addition to measures of commonly reported negative problems resulting from cannabis use. We compared those who met a proxy measure of DSM-IV-TR criteria for cannabis dependence (N = 1111) to those who did not meet the criteria (N = 1770). Cannabis dependent subjects consumed greater amounts of cannabis, alcohol, and a variety of other drugs. They also had lower levels of motivation, happiness, and satisfaction with life, with higher levels of depression and respiratory symptoms.

**Conclusion:**

Although all of our subjects reported daily use, only those meeting proxy criteria for cannabis dependence reported significant associated problems. Our data suggest that dependence need not arise from daily use, but consuming larger amounts of cannabis and other drugs undoubtedly increases problems.

## Background

More people consume cannabis than any other illicit substance in America. In 2000, 76 percent of all current illicit drug users (14 million people) consumed cannabis [1]. Anthony, Warner, and Kessler [2] estimate that cannabis produces dependence in 9.1 percent of people who ever use cannabis in their lifetime. Therefore, not all cannabis users meet the DSM-IV-TR criteria for dependence. The causes of cannabis dependence remain poorly understood. One theory states that the frequency of cannabis use is associated with dependence [3,4]. In a study of the relationship between frequency and quantity of cannabis use and dependence, frequency of use was shown to be linearly associated with the probability of dependence and was a stronger predictor than quantity of use [5]. The 2003 National Survey on Drug Use and Health demonstrated that only 13.5 percent of non-daily users were cannabis-dependent, as compared to 39.2 percent of daily users [6]. Although daily users in this national survey had higher rates of dependence, more than half of the subjects failed to meet the criteria. Thus, daily use is no guarantee of dependence.

The quantity of cannabis consumption may determine dependence. In 450 cannabis users who met the criteria for dependence, Stephens, Babor, Kadden, Miller, and The Marijuana Treatment Project Research Group [7] found that the typical participant smoked cannabis almost daily and consumed nearly three joints per day and almost half an ounce of cannabis per week. They also reported getting high more than four times per day and felt high for approximately seven hours each day. On average, these subjects reported experiencing upwards of five dependence symptoms, demonstrating that using larger amounts of cannabis and feeling high for longer periods may contribute to cannabis dependence.

Comorbid use of alcohol may also increase cannabis dependence. Frequent or heavy alcohol use in conjunction with cannabis increases the probability of dependence [8]. Cannabis dependent users are more likely to meet the criteria for alcohol dependence [9], as well as report consuming more alcoholic drinks in one sitting [10]. Bell, Wechsler, and Johnston [11] also observed that students who were binge drinkers were far more likely to use cannabis than students who did not binge drink. Although it is difficult to determine the exact causal relationship between alcohol and cannabis use, alcohol does appear to moderate the association between cannabis use and dependence.

A user's age may also determine dependence. Adolescents, particularly those with an early onset of cannabis use, are more susceptible to cannabis dependence [12,13]. Cannabis users under the age of 17 are 3.44 times more at risk for meeting three dependence symptoms than are users over the age of 26 [14].

In addition to exploring the causes of cannabis dependence, much of the current literature on cannabis has sought to describe the characteristics of heavy users. Several studies have noted an association between cannabis use and lower educational attainment [15,16]. Fergusson, Horwood, and Beautrais [17] analyzed a 25-year longitudinal study and demonstrated that at age 16, those who had used cannabis on more than 100 occasions were nearly six times as likely to leave school without graduating as those who had never used cannabis. In the same sample, students at the age of 20 who had never used cannabis were four and a half times more likely to receive a college degree than students who had used cannabis more than 100 times. In contrast, Reilly, Didcott, Swift, and Hall [18] found a sample of long-term Australian cannabis users to be well-educated, with 62 percent of the participants having obtained higher education after leaving school.

A correlation has been posited between cannabis use and lack of motivation and depression. McGlothlin and West [19] describe an "amotivational syndrome" amongst heavy cannabis users entailing apathy, loss of effectiveness, and a diminished ability to concentrate, follow routines, and successfully master new material. Similarly, Musty and Kaback [20] detected amotivational symptoms in a sample of heavy cannabis users, but attributed the behavior instead to coexisting depressive symptoms. Research on the affective states of cannabis users has suggested that cannabis abusers are more likely than non-abusers to report feeling depressed [21] and heavy cannabis use may actually increase depressive symptoms [22]. Furthermore, individuals with cannabis dependence are at greater risk of suicide attempts, perhaps due to a comorbid mood disorder [23]. Despite these findings, other work does not support a link between cannabis use and depression [24], nor an association between cannabis use and lack of motivation [25-27].

In addition, both current and heavy users have been shown to consistently report lower levels of satisfaction with quality of life than control subjects [28]. Stephens et al. [7] similarly demonstrated that in a sample of cannabis dependent individuals, 90 percent felt bad about their use and almost 75 percent had lowered self-esteem. The authors speculate that people who seek treatment are primarily motivated by these feelings that stem from dependence. This result illustrates that many heavy cannabis users report a decreased quality of life and well-being.

Finally, cannabis dependent users have been shown to have a significantly increased likelihood of reporting a wide range of respiratory symptoms and exhibiting decreased lung functioning [29]. In a nationally representative sample of 6728 adults, cannabis use was associated with a number of problems including bronchitis, coughing, phlegm production, and wheezing, even after controlling for age, tobacco use, and asthma [30]. Given that only 16 percent of the sample used cannabis on a daily basis, it is likely that the range of respiratory problems is even greater in a cannabis dependent population. On the contrary, Tashkin, Simmons, Sherrill, and Coulson [31] found no evidence of habitual cannabis use contributing to decreased lung functioning or chronic obstructive pulmonary disease.

The current study seeks to examine if the discrepancies surrounding the research on cannabis use can be resolved by separating out those users who meet the DSM-IV-TR criteria for cannabis dependence. We aim to determine possible links to cannabis dependence and to demonstrate whether dependent cannabis users are more likely to report these negative aspects of cannabis use than non-dependent users, even when both groups are daily users.

## Methods

### Participants

Participants responded to an email request. In an effort to target frequent cannabis users, three organizations committed to altering drug laws (The Marijuana Policy Project, The National Organization for the Reform of Marijuana Laws, and The Drug Policy Alliance) were asked to send a query to their mailing lists for participation in a survey in return for the chance to win a cash prize. Participants were also asked to forward the link to their friends. Approximately 9,000 people replied.

We focus on those who currently used cannabis daily. The 2881 people who qualified included 1842 men (63.9%) and 1039 women (36.1%). Ages ranged from 18 to 88 (*M *= 33.03, *SD *= 12.72). Education ranged from some high school to advanced degrees, with a median of some college without a bachelor's degree. Median income was $20,000 to $40,000 per year. Respondents were primarily Caucasian (87%), but included African Americans (1%), Native Americans (3%), Asians (1%) Latinos (1%), and people of mixed race (5%). Two percent of participants preferred not to answer. Those who used cannabis began at a mean age of 16.8 (*SD *= 4.3).

### Procedure

The protocol and informed consent were approved by a local Institutional Review Board. Upon beginning the survey, subjects were informed of the nature of the study and provided consent. Participants were informed that they would be responding to questions regarding their drug use, as well as indices of quality of life. Brief questionnaires were utilized to assess many of our variables of interest in order to increase response and retention rate.

#### Cannabis use

Participants reported if they ever used cannabis in their lifetime. Those who said they had ever used were also asked if they had used in the previous month. Those who had used in the previous month were asked how many days per week they used on average. All participants in this study reported using 7 days per week. Although estimating quantity of consumption is difficult for cannabis, they also reported the number of joints they consumed per week. Furthermore, participants responded to questions based on the DSM-IV-TR criteria for substance dependence (see Table 1). These self-reported symptoms served as a proxy measure for cannabis dependence. Each item required the participant to respond affirmatively or negatively to a question regarding their experience with a particular dependence symptom within the past 12 months. Subjects who endorsed three or more of the 7 items were considered to be cannabis dependent, as designated by the DSM-IV-TR criteria for substance dependence [32].

**Table 1:** Proxy cannabis dependence questions

Item
1. Do you use cannabis much more often or in greater amounts than you intended?
2. Did you want to or try to cut down on your cannabis use but find that you could not?
3. Did your cannabis use keep you from going to work or school, or engaging in recreational activities?
4. Have you experienced a month or more of spending a great deal of time getting cannabis, using it, or recovering from its effects?
5. Did cannabis cause you emotional or psychological problems, such as feeling uninterested in things, feeling depressed, feeling suspicious, feeling paranoid, or having strange ideas?
6. Have you developed a tolerance to cannabis so that the same amount of drug had less effect than before?
7. Has cannabis caused you to experience withdrawal symptoms?

#### Alcohol consumption

Participants reported their average number of standard drinks consumed on an occasion and the frequency with which they drank alcohol per week.

#### Other drug use

Participants reported the number of tobacco cigarettes they consumed each day as well as whether or not they had used any of the following drugs in the last year: inhalants, methamphetamine, speed, cocaine, crack cocaine, ecstasy, psilocybin, LSD, and heroin.

#### Cannabis associated problems

Participants responded to nineteen questions from the Marijuana Problems Questionnaire (MPS) regarding problems associated with their cannabis use in the past 90 days on a six-point scale (i.e. 0 = no problem, 5 = serious problem) [33]. Examples of included items are: Problems in your family; Problems between you and your partner; Missing days at work or class; Withdrawal Symptoms; To feel bad about your use.

#### Respiratory symptoms

Six questions appeared based on previous studies of cannabis's impact on respiratory symptoms. These included: Do you usually have a cough? Does your chest sound wheezy or whistling other than from colds? Are you troubled by shortness of breath when hurrying on the level ground or walking up a slight hill? Do you have to walk slower than most people your own age on the level ground because of breathlessness? Do you cough up phlegm in the morning? Do you wake up at night with tightness in your chest? [29].

#### Satisfaction with Life

Participants completed Diener's 5-item Satisfaction With Life Scale [34]. Internal consistency was .85.

#### Happiness

Participants completed the Happiness Scale, a 4-item report of subjective happiness [35]. Internal consistency was .86

#### Motivation

Respondents completed items from the Apathy Evaluation Scale (AES) [36]. Participants responded to 12 statements regarding their own feelings of motivation on a four-point scale (e.g. Not At All; Slightly; Somewhat; Very Much). Eight items that accounted for the most variance in the scale were selected. Additional items were added based on face validity. Items included: I get things done during the day; getting things done during the day is important to me; I approach life with intensity; Seeing a job through to the end is important to me; Getting things done on my own is important to me; I have initiative; I have motivation; I set goals for myself; I don't follow through on my plans (reverse coded); I have some interesting projects I'm working on; I'm pretty productive most of the time; I am interested in things. The twelve items possessed sound internal consistency (Cronbach's α =.82). Similar measures of apathy have been used successfully in previous studies of substance use and motivation, showing increased apathy among the cocaine dependent [37].

#### Depression

The Center for Epidemiologic Studies Depression scale (CES-D) [38], a popular self-report measure designed to explore depressive symptoms in the general population, contains 20 items. The CES-D asks respondents to indicate the frequency or duration of specific symptoms associated with depression (e.g., "I had crying spells") during the previous week. Internal consistency was .90.

## Results

Of all the participants, 1111 subjects met the criteria for proxy dependence (DEP), while 1770 subjects were non-dependent (N-DEP). Missing data was found for many of the variables of interest. However, given that no more than 5% of participants failed to respond on each of the variables, these cases were not removed since they should not impact our findings and retaining these individuals will increase our overall power.

Independent samples t-tests revealed that cannabis dependent individuals (*M *= 27.895, *SD *= 10.63), relative to non-dependent volunteers (*M *= 36.34, *SD *= 12.88), were significantly younger [*t *(2839) = 18.18; *P *< .001], and had fewer years of schooling (DEP: *M *= 3.43, *SD *= 1.23; N-DEP: *M *= 3.59, *SD *= 1.41), [*t *(2870) = 3.18; *P *< .01]. Table 2 shows that cannabis dependent participants reported smoking a greater number of joints per week, using a larger amount of cannabis by quarter ounce per month, feeling higher on average during cannabis consumption, experiencing a greater maximum high, averaging a greater number of alcoholic drinks per drinking occasion, and consuming more alcoholic drinks per week than non-dependent daily users. In addition, Table 2 shows that happiness, satisfaction with life, and motivation are decreased and depression is increased in dependent individuals. Furthermore, those cannabis dependent individuals experienced more respiratory and additional problems associated with cannabis use than did non-dependent users. Since a large number of subjects consumed tobacco cigarettes in addition to cannabis (DEP: *n *= 837; N-DEP: *n *= 1398), an ANCOVA was performed to covary out cigarette use. Cannabis dependence continued to predict respiratory problems, even after cigarettes were accounted for (*F *(1, 2879) = 133.68, *p *< .001). Because cigarette use was a significant predictor, we are reporting the covariate corrected means of respiratory symptoms for the cannabis groups (DEP: *M *= 1.29, *SD *= 1.20; N-DEP: *M = *.77, *SD *= 1.18).

**Table 2:** Comparison of cannabis dependent and non-dependent participants on indices of cannabis use and related problems

Index	Dependent		Non-Dependent		
	*n*	Mean (SD)	*n*	Mean (SD)	*t*
Joints/Week	1083	16.58 (15.18)	1743	14.14 (13.61)	-4.32*
Cannabis/Month	1105	4.75 (2.09)	1764	4.18 (2.13)	-7.09*
Average High	1107	4.06 (1.23)	1762	3.51 (1.28)	-11.35*
Maximum High	1109	5.47 (1.03)	1763	4.95 (1.37)	-11.59*
Alcohol Drinks/Occasion	1109	4.97 (4.40)	1766	3.28 (3.64)	-10.68*
Alcohol Drinks/Week	1109	11.49 (17.61)	1768	7.30 (14.22)	-6.68*
Respiratory Problems	1111	1.28 (1.33)	1770	0.77 (1.12)	-10.61*
Cannabis-Related Problems	1111	12.08 (11.00)	1770	4.21 (5.54)	-22.14*
Happiness	1105	15.25 (3.71)	1756	16.23 (3.47)	7.01*
Satisfaction With Life	1109	18.92 (5.45)	1758	19.89 (5.28)	4.69*
Motivation	1092	49.60 (4.70)	1753	51.56 (4.03)	11.44*
Depression	1059	13.23 (9.69)	1700	8.45 (8.19)	-13.35*

*p < .001

Chi-square tests revealed higher use of other illicit drugs in the dependent group. Table 3 shows that cannabis dependent subjects were more likely to have used inhalants, methamphetamine, speed, cocaine, crack cocaine, ecstasy, psilocybin, and LSD in the past year than non-dependent users.

**Table 3:** Proportions of past year illicit drug use by dependent and non-dependent cannabis users

Illicit Drug	Dependent % Reported Using	Non-Dependent % Reported Using
Inhalants	10.98	4.08**
Methamphetamine	10.44	7.29**
Speed	18.45	7.63**
Cocaine	33.21	17.34**
Crack Cocaine	3.78	2.32*
Ecstasy	18.09	10.00**
Psilocybin	47.07	25.71**
LSD	15.48	9.49**
Heroin	0.99	0.90

*Note. *Significance determined using χ^2 ^test with *df *= 1.

* *p *< .05 ** *p *< .01

## Discussion

We examined how behaviors and problems differed between dependent and non-dependent daily cannabis users, using a proxy measure of dependence based upon the DSM-IV-TR criteria. The findings from this study provide insight into the inconsistencies seen in the cannabis literature. Various traits and problems are seen more often in some cannabis users compared to others, and the data show that these differences can be attributed to whether or not the user is cannabis dependent. The results demonstrate that dependent daily cannabis users are more likely to be younger and less educated than non-dependent daily users. Although frequency of use was the same between the two groups, dependent users consumed a larger amount of cannabis per week and per month, and reported getting higher and achieving a greater maximum high than non-dependent users. In addition, alcohol consumption differed between the groups, with dependent users ingesting the greatest number of alcoholic beverages per occasion and per week. Furthermore, dependent cannabis users were more likely to have used a variety of illicit drugs within the past year than were non-dependent users.

The two groups also differed on ratings related to mood. Cannabis dependence was associated with higher levels of depression, and lower levels of motivation, satisfaction with life, and happiness. Dependent users additionally suffered a greater number of respiratory problems, even when cigarette smoking was accounted for. Finally, cannabis dependent individuals more frequently reported additional cannabis-related problems than did non-dependent subjects. These results are consistent with findings that cannabis dependent individuals, particularly those who experience tolerance and withdrawal, are more likely to self-report problems related to cannabis use [39,40].

There are some limitations to this study relating to sampling, Internet survey methodology, and the use of a proxy measure for diagnosis. Due to recruitment through drug policy organizations, our subjects were possibly disinclined to report negative symptoms related to cannabis use. Therefore, our data may be an underestimate of true cannabis-related symptoms, yet we were still able to discover differences between the two groups. In addition, we saw a full range of cannabis dependence scores (0 – 8) and a wide range of CESD scores (0 – 55; maximum score is 60) [38]. Thus, it is likely that many participants were in fact willing to admit to negative consequences associated with cannabis use.

Additionally, although Internet-based data collection allows us to collect data from a nationwide sample and from drug users who would be unwilling to travel to our laboratory, it is unclear whether those who suffer the worst negative consequences of drugs are willing to disclose all symptoms and the full extent of their drug use. However, in a study by Wang et al. [41], students were more likely to admit to substance use in a web-based questionnaire than on a paper-and-pencil questionnaire. Therefore, it is probable that participants were more willing to admit to drug use, perhaps due to the remote and anonymous nature of the Internet. Additionally, participants in this study were primarily Caucasian, male, and relatively young, which limits the generalizability of our results. In addition, some individuals with lower education or socioeconomic status may not have access to the Internet, excluding them from the study. Future research should observe differences between dependent and non-dependent daily cannabis users in a more diverse population.

Use of short self-report questionnaires further limits the conclusions that can be drawn from this study. Though these measures were chosen due to their brevity, their subjectivity in nature allows us to conclude only that cannabis dependent individuals view themselves as less happy and less satisfied with life. No objective markers of quality of life such as employment status, social roles, or activities have been examined, and should be made a focus in future research. Additionally, the reliability of self-report is called into question due to the fact that many participants were long-term cannabis users. It is possible that prolonged drug use may have produced neurophysiological changes, resulting in errors in report. A recent report by Degenhardt and Hall [42] has established early cannabis use as a risk factor for later psychosis. Though this study was not designed to assess for severe psychopathology, it is plausible that a subset of our participants may have experienced symptoms of psychosis, influencing the reliability of our results.

We also want to stress the limitation of this study resulting from the proxy measure of cannabis dependence. While participants responded to questions specific to each of the 7 criteria for substance dependence, diagnostic data were not obtained from an established method designed to evaluate these criteria. Therefore, a definitive diagnosis of cannabis dependence cannot be obtained. However, the items used as a proxy for dependence strictly adhered to the criteria in the DSM-IV-TR, so we have reason to believe that this assessment and a structured interview would produce comparable results. In fact, it is more likely that we have an underestimate of cannabis dependent subjects since further probing of the subjects' responses was absent due to the computer-based survey.

Although our data suggest a link between a proxy for cannabis dependence and the occurrence of various problems and experiences, it is difficult to know whether cannabis dependence led to, or was a result of these differences. A study by Von Sydow, Lied, Pfister, Hofler, and Wittchen [43] determined that cannabis dependence was best predicted by early parental death, low socio-economic status, and additional illicit drug use. It is possible that in our sample, past year drug use may have been a causal factor leading to cannabis dependence, whereas the other variables could have resulted from this dependence. While the actual direction remains unknown, there is clearly a relationship between these variables.

These results have important implications for the screening and treatment of cannabis dependence. It is important for treatment providers to be aware of all of the potential problems associated with cannabis dependence, as well as the potential comorbid alcohol and drug use. Assessments of cannabis use should be conducted in depressed persons, as current or prior use of the drug may be contributing to the current mood state. In addition, cannabis users should be made conscious of the fact that increased cannabis consumption is likely to produce or enhance many problems and undesirable mood states. Education on recognition of cannabis dependence and cannabis-induced problems is imperative in order to decrease the troubles noted in this population. Public knowledge about the negative effects of cannabis use may assist in motivating treatment amongst many users who are currently resistant or ambivalent towards change.

The notion that cannabis use in moderation may not be problematic, especially if alcohol consumption is also limited, might be particularly salient to those users who recognize problems associated with cannabis use, but who are not motivated to completely abstain. The subjects in our study who did not meet the criteria for proxy dependence consumed smaller quantities of both cannabis and alcohol, while also reporting fewer mood and behavioral problems, even though they were daily users. Therefore, many users may have the ability to consume cannabis in limited quantities with few associated problems. Media campaigns relaying the message that there is such as thing as too much cannabis use, and edifying users about the nature of these associated problems, may assist in directing cannabis dependent individuals toward exploring treatment options.

## Competing interests

The authors declare that they have no financial competing interests to report. ME works for organizations devoted to altering cannabis policy.

## Authors' contributions

AL undertook data analysis and drafted the manuscript. ME had the original idea for the study, provided overall project management, supervised AL as she undertook the process of data analysis, and provided input to subsequent drafts of the manuscript. All of the authors read and approved the final manuscript.
